# A Review of Manganese-Oxidizing Bacteria (MnOB): Applications, Future Concerns, and Challenges

**DOI:** 10.3390/ijerph20021272

**Published:** 2023-01-10

**Authors:** Yanan Cai, Kun Yang, Chaochao Qiu, Yunze Bi, Bowen Tian, Xuejun Bi

**Affiliations:** School of Environmental and Municipal Engineering, Qingdao University of Technology, Qingdao 266520, China

**Keywords:** manganese-oxidizing bacteria, biofilter, groundwater, Mn removal, biogenic Mn oxides

## Abstract

Groundwater serving as a drinking water resource usually contains manganese ions (Mn^2+^) that exceed drinking standards. Based on the Mn biogeochemical cycle at the hydrosphere scale, bioprocesses consisting of aeration, biofiltration, and disinfection are well known as a cost-effective and environmentally friendly ecotechnology for removing Mn^2+^. The design of aeration and biofiltration units, which are critical components, is significantly influenced by coexisting iron and ammonia in groundwater; however, there is no unified standard for optimizing bioprocess operation. In addition to the groundwater purification, it was also found that manganese-oxidizing bacteria (MnOB)-derived biogenic Mn oxides (bioMnO_x_), a by-product, have a low crystallinity and a relatively high specific surface area; the MnOB supplied with Mn^2+^ can be developed for contaminated water remediation. As a result, according to previous studies, this paper summarized and provided operational suggestions for the removal of Mn^2+^ from groundwater. This review also anticipated challenges and future concerns, as well as opportunities for bioMnO_x_ applications. These could improve our understanding of the MnOB group and its practical applications.

## 1. Introduction

The elements on Earth have their own biogeochemical cycle pathways on the biosphere scale, and they may interact with one another [1,2]. These cycles are critical to the water ecosystem [1]. For example, the effective circulation of nitrogen elements from nitrate to nitrogen gas in the hydrosphere can prevent eutrophication. This implies that water or wastewater treatment technologies can be developed using the redox cycles [3].

**Figure 1:**
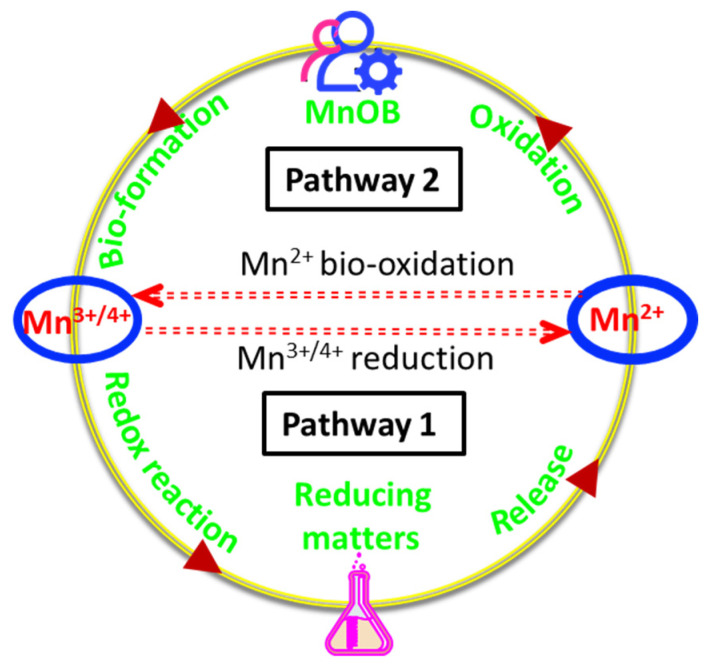
The diagram of the Mn cycle mediated by MnOB and reducing matters.

It is critical to understand the macroscopic manganese (Mn) cycle. The element Mn is abundant in the earth’s crust. It is ranked fifth among metal elements and second only to iron among transition metals [4]. The Mn element has 11 valence states, ranging from −3 to +7; however, only +2, +3, and +4 are naturally present in the biosphere [5]. Dissolved Mn^2+^ can be found in a variety of water bodies, including surface water (rivers and lakes) [6,7,8], groundwater [9,10], and oceans [11]. Because free or dissolved Mn^3+^ is extremely unstable, it is easily transformed into Mn^2+^ or Mn^4+^ through disproportionation [12], or an oxyhydroxide (MnOOH) is formed. Mn oxides (MnO_x_) are typically found in sediments and Mn ores, appearing in a complicated form of Mn^4+^, Mn^3+^, and Mn^2+^. The interconversion of Mn^2+^, Mn^3+^, and Mn^4+^, as shown in Figure 1, forms the biogeochemical cycle of Mn at the hydrosphere scale. MnO_x_ is the most powerful natural oxidant in pathway I, with typical redox potentials of 1.23 V [13] and 1.51 V [14], respectively (Equations (1) and (2)). As a result, a redox reaction between MnO_x_ and reducing substances (such as organic matter in sediments) might occur, resulting in the release of Mn^2+^ into the surrounding aquatic environment. As in pathway II, the dissolved Mn^2+^ will be re-oxidized into MnO_x_ by manganese-oxidizing bacteria (MnOB) and oxygen. In comparison to oxygen, however, it appears that the MnOB group dominates pathway II due to a greater oxidation rate [15], which is several orders of magnitude higher [16].

MnO_2_(s) + 4H^+^ + 2e^−^ = Mn^2+^ + 2H_2_O     *E*^0^ = 1.23 V (1)

MnOOH(s) + 3H^+^ + e^−^ = Mn^2+^ + 2H_2_O     *E*^0^ = 1.51 V (2)

**Figure 2:**
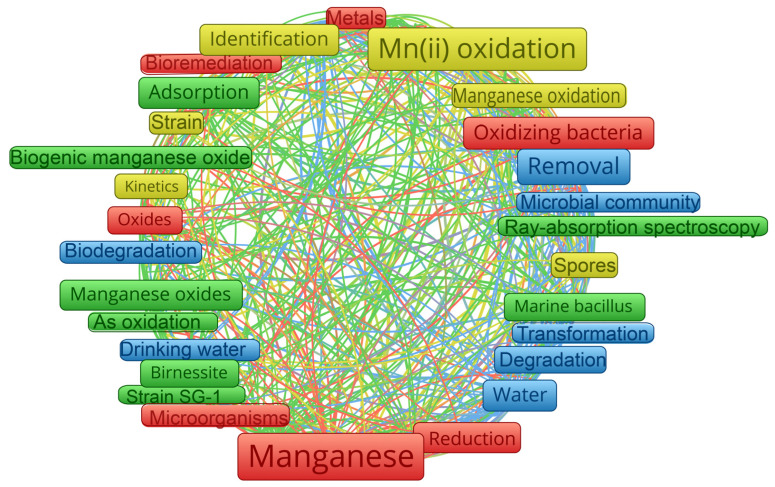
Network view map of 859 publications in the literature, generated by using VOSviewer.

MnOB have received attention throughout the past 20 years. The number of publications retrieved with the keywords “manganese oxidizing bacteria or bioMnO_x_” from well-known publishers is increasing. VOSviewer software (version 1.6.18) was used to process Web of Science Core Collection data. The most common topics, as can be seen in Figure 2, are the oxidation and removal of Mn^2+^, the characterization and use of bioMnO_x_, the identification of MnOB, and multicopper oxidases. On the one hand, compared to the removal of Mn^2+^ from groundwater by chemical oxidants such as chlorine, potassium permanganate, and ozone, the oxidation of Mn^2+^ to Mn^3+^ and Mn^4+^ using MnOB without any chemicals offers a low-cost and environmentally friendly method of removing Mn^2+^ for drinking purposes. However, there are currently no specifications for the design or operation of bioprocesses that can be referenced as a guide, which could result in a number of operational issues, such as excessive energy use. Moreover, in addition to groundwater purification, because the cycle in Figure 1 is regulated by numerous environmental factors [17], organic compounds such as antibiotics [18] and dyes [19] can be absorbed and degraded by biogenic MnO_x_ (bioMnO_x_). It possesses advantages over chemical approaches for the treatment of contaminated water since it is a natural biosorbent and oxidant. However, the release of Mn^2+^ has been demonstrated during the degradation of organic compounds, which can lead to secondary pollution or a decrease in reactivity.

Therefore, the properties of MnOB and bioMnO_x_ were investigated in this paper. The bioprocess for removing Mn^2+^ from groundwater using functional MnOB was evaluated, and its operational suggestions were also concluded. Moreover, this paper suggests a suitable process technique in accordance with the applications of bioMnO_x_ in bioremediation. It is possible to provide an in-depth comprehension of foundation research and engineering construction.

## 2. The MnOB Group

### 2.1. The Phylogenetic Diversity of MnOB

Microorganisms that can oxidize Mn^2+^ into insoluble bioMnO_x_ are known as Mn oxidizing organisms, and these organisms include bacteria, eukaryotes, fungi, and others [20]. As can be observed, the MnOB group has received the greatest attention, most likely because of its significant contribution to Mn^2+^ oxidation. Although MnOB can be isolated, most of them are still impossible to distinguish through pure culture. This can be attributed to selection differences in the medium [20], spontaneous loss of Mn oxidation ability in the laboratory [21], or to the fact that some MnOB strains do not oxidize Mn^2+^ alone [22]. *Leptothrix*, *Pseudomonas*, *Hyphomicrobium*, and *Bacillus* have so far been recognized as typical MnOB genera [15,20,23,24]. According to Hope et al. [25], *Leptothrix* is a dominant MnOB in Mn removal biofilters. Burger et al. [26] found that only one of four full-scale water supply plants had *Leptothrix* in its biofilters, whereas the other three plants that tested positive for biological Mn removal did not. Cheng et al. [27] demonstrated that *Gallionella* and *Crenothrix* were the dominant MnOB in a pilot-scale biofilter. Yang et al. [28] indicated that in addition to *Pseudomonas*, *Hyphomicrobium*, and *Bacillus*, the MnOB in a biofilter were also dominated by *Acinetobacter*, *Pedomicrobium*, *Hydrogenophaga*, and *Microbacterium*. This implies that the well-known, typical MnOB may not exist in a Mn-rich environment. Table 1 lists the MnOB group identified in various Mn environments. In addition to the typical MnOB genera mentioned above, there are a number of additional MnOB found in rivers, lakes, oceans, groundwater, and Mn deposits. These bacteria belong to a variety of phyla; isolated MnOB differ from one another even in the same water environment. Furthermore, some MnOB remain unrecognized. According to [22], the majority of the MnOB members in a culture belong to the phylum Nitrospirae but are distantly related to *Nitrospira* and *Leptospirillum*.

In general, highly abundant bacteria in the Mn environment are considered MnOB [29,30,31], but it is crucial to clearly identify MnOB from other functional bacteria. This is because the Mn cycle frequently occurs in conjunction with other cycles of substances present in the Mn environment, resulting in the coexistence of functional bacteria. The results indicated that functional bacteria involved with Mn/Fe oxidation–reduction [32], nitrogen transformation [33], and sulfur oxidation [34] can be discovered in the Mn environment at the same time. As previously observed, these functional bacteria are likely to contribute significantly to the overall bacterial community or have a positive relationship to MnOB [31,34]. Therefore, it is essential to accurately identify the MnOB group.

**Table 1:** The MnOB-related group detected from various Mn environments.

Environmental Conditions	The MnOB Group	References
River	*Bacillus*, *Pseudomonas*, *Sphingomonas*, *Hyphomicrobium*, *Cyanobacteria*	[31]
	*Microbacterium*, *Agromyces*, *Mycobacterium*, *Arthrobacter*, *Pseudomonas*, *Burkholderiales*	[35]
	*Aurantimonas*, *Rhodobacter*, *Bacillus*, *Shewanella*	[36]
Lake	*Metallogenium*, *Leptothrix*, *Siderocapsa*, *Naumaniella*, *Bacillus*, *Pseudomonas*	[37]
	*Bacillus*, *Pseudomonas*, *Afipia*	[35]
Well	*Acinetobacter* sp., *Bacillus*, *Sphingobacterium* sp.	[38]
	*Pseudomonas*, *Burkholderiales*	[35]
Mn deposits	*Bacillus*, *Exiguobacterium*, *Staphylococcus*, *Brevibacterium*, *Alcanivorax* sp.	[39]
	*Hyphomicrobium*, *Leptolyngbya*	[40]
	*Sphingomonas*, *Flavobacterium*, *Janthinobacterium*, *Acinetobacter*	[30]
Seawater	*Bacillus* sp. FF-1	[41]
	*Citreicella manganoxidans* sp. nov.	[42]
	*Marinobacter manganoxydans* MnI7-9	[43]
Activated sludge	*Brevibacillus*	[44]
	*Serratia marcescens*	[45]
Biofilter	*Pseudoalteromonas* sp.	[46]
	*Flavobacterium*, *Brevundimonas*, *Stenotrophomonas*	[47]
	*Leptothrix*, *Pseudomonas*, *Methylibium*	[18]
Drinking water system	*Bacillus*	[35]
	*Lysinibacillus*, *Bacillus*, *Pseudomonas*, *Brevundimonas*	[48]

### 2.2. MnOB Recognition

The identification of MnOB is intended to confirm their oxidation capacity or taxonomic status. After the Mn^2+^ is oxidized, the insoluble bioMnO_x_, which appears as black–brown, is often coated on the surface of MnOB [15,49]. As a result, the most direct method of determining oxidation ability is morphological observation. For example, in a biofilter packed with quartz sand and continually fed with Mn^2+^, MnOB were successfully enhanced when the sand transformed from white to black–brown [18]. The approach can also be used to identify MnOB in a culture medium. However, when Fe^2+^ and Mn^2+^ coexist in water or a culture medium, MnOB must be distinguished from iron oxidizing bacteria, as the biogenic iron oxides may coat the bacterial surface and appear similar to bioMnO_x_. In such cases, the reductive Leucoberbelin blue (LBB) dye, which interacts with Mn oxides to form a blue solution, can be utilized for further chemical detection.

The most common genotypic approach for determining the taxonomic status of MnOB is 16S rRNA gene sequence technology, which includes DNA extraction, polymerase chain reaction (PCR) amplification, and sequencing. The primers used in the PCR should be specific for a particular MnOB genus. However, it is currently challenging to design enough specific primers to detect the numerous MnOB groups. This explains why universal primers are commonly utilized in most investigations. The universal primers allow researchers to explore the most relevant or dominant MnOB communities in a system [19,34], but this is dependent on the sequences deposited in the database.

Moreover, the process by which MnOB oxidize Mn^2+^ is unclear. It is well accepted that the oxidation of Mn^2+^ is an extracellular enzymatic reaction employing various multicopper oxidases (MCOs). Several genes have been identified as being involved in the coding of MCOs for Mn^2+^ oxidation, including *mofA* in *L*. *discophora* SS-1, genes in the *ccm* operon of *P*. *putida* MnB1, *mnxA*, *B*, *C*, *D*, *E*, *F*, and *G* in *P*. *putida* SG-1; *cumA* in *P*. *putida* GB-1 [5]; *moxA* in *Pedomicrobium* sp. ACM 3067; *mokA* in *Lysinibacillus* strain MK-1; *cotA* in *Bacillus pumilus* WH4 [17]; *cueO* amplified from *Escherichia coli* [50]; and *copA* in *B*. *panacihumi* MK-8 [51]. Therefore, primers based on these functional proteins can be used to identify MnOB with the same protein gene, but further classification of MnOB still requires the assistance of 16S rRNA gene sequencing.

## 3. Engineering Application of MnOB for Mn^2+^ Removal

### 3.1. The Bioprocess

**Figure 3:**
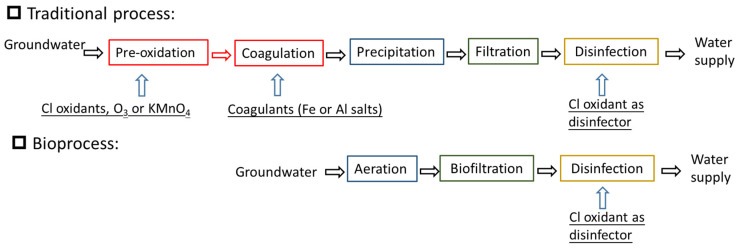
Processes designed for removal of Mn^2+^ from groundwater.

Groundwater, which serves as a source of drinking water, usually contains Mn^2+^ because of the anaerobic environment. The Mn^2+^ concentration should be controlled below 0.10 mg/L or 0.05 mg/L [52]. As illustrated in Figure 1, MnOB can be used to remove Mn^2+^ from groundwater. Figure 3 shows the bioprocess, in which a sand biofilter was built as the functional unit, for removing Mn^2+^ from groundwater. This bioprocess is simpler than conventional drinking water purification processes (coagulation, precipitation, filtration, and disinfection) and no chemical oxidants are required. Li et al. [53] estimated that it might save CNY 50 million in construction costs and CNY 12,000 in daily operation and maintenance costs for a 12 × 10^4^ m^3^/d water supply plant. Given that switching from a non-biological to a biological process can greatly enhance processing capacity and save operational costs by up to 80% [10], this provides a technical transformation strategy for the groundwater treatment facility.

### 3.2. The Aeration Unit

Because iron (Fe) and Mn are frequently found together in groundwater, and because the problems they cause are comparable and can be removed simultaneously by biofilters, they are discussed together. Bio-oxidation of Fe^2+^ or Mn^2+^ requires very little dissolved oxygen (DO). According to Equations (3)–(5), 0.14 mg and 0.29 mg DO are required per mg of Fe^2+^ and Mn^2+^, respectively. Therefore, there are no strict limits on the procedure as long as the aeration unit provides appropriate DO. In general, a simple aeration technique, such as falling water aeration, can provide the necessary level of DO. It was found that groundwater with high concentrations of Fe^2+^ ~ 15 mg/L and Mn^2+^ ~ 2.0 mg/L could be efficiently treated under a low DO of 4–5 mg/L [54].

4Fe^2+^ + O_2_ → 4Fe^3+^ +2O^2−^ (3)

2Mn^2+^ + O_2_ → 2Mn^4+^ +2O^2−^ (4)

[O_2_] = 0.14[Fe^2+^] + 0.29[Mn^2+^] (5)

High aeration is thought to increase pH, hence accelerating the oxidation of Fe^2+^ or Mn^2+^ [55]. It was discovered by Hoyland et al. [56], however, that biofilter columns operating at pH 6.3 and 6.7 began to remove Mn^2+^ earlier than those operating at higher pH. This is to be expected, given that inorganic carbon is a carbon source for MnOB [22]; yet, high aeration removes more inorganic carbon from groundwater. Excessive aeration appears to be unnecessary because the MnOB is active at both weak and neutral pH conditions. This should be taken into account when designing the aeration unit.

In addition to Fe^2+^ and Mn^2+^, ammonia may be present in groundwater. The nitrification process by which nitrifiers convert ammonia to nitrate dominates ammonia removal. As the nitrification process takes 4.57 mg of DO per mg of ammonia, the presence of ammonia pressures the aeration unit; however, the actual demand for DO was lower than the theoretical calculation estimate. This demonstrated that autotrophic anammox bacteria, which do not require DO, contribute to the conversion of ammonia to nitrogen gas and nitrate, accounting for 48.5%, 46.6% [57], and 15.92% [28] of the removal, respectively. This provides an optimization design proposal for the aeration unit. Furthermore, an additional aerator should be installed at the bottom of the filter bed if the aeration unit fails to supply enough DO. This constant aeration may weaken the interception ability of the filter bed, resulting in metal oxide residues in the effluent. To ensure the quality of the water, a second filtration step is required after the biofilters.

### 3.3. The Biofilter

The biofilter is the central component of the bioprocess, and its start-up and performance are of particular interest. The main drawback of biofilters appears to be their lengthy start-up phase, during which Mn^2+^ is ineffectively removed. This phase might last for weeks or months. According to previous studies, the start-up time could be shortened by using backwashing sludge or mature biofilter-supporting materials as inocula [30]. Moreover, during the maturation stage of biofilters, the usage of special filtering media with Mn^2+^ adsorption capacity can ensure the quality of the water [58]. The biological removal of Mn^2+^ has been widely employed all around the world. It shows that the biofilter can still be operated to remove Mn^2+^, even at low temperatures of 4 °C [4], 3–4 °C [59,60], and 8–14.8 °C [61].

The biofilters are frequently designed to operate at a lower rate of ~2 m/h at start-up period [54]. Actually, they have a very high treatment load following the start-up period. Štembal et al. [62] observed that at an average Mn^2+^ concentration of 1.06 mg/L, the biofilter’s filtration rate can reach 24 m/h. Cheng et al. [63] discovered that when fed 8 °C groundwater containing total Fe of 5–10 mg/L and ammonia of 0.9–1.3 mg/L, a pilot-scale biofilter working at 6 m/h could tolerate a maximum Mn^2+^ concentration of about 10 mg/L. Fe^2+^ and Mn^2+^ can be removed simultaneously in a one-stage biofilter, where Fe^2+^ is removed in the top filter layer and Mn^2+^ is bio-oxidized and removed in the lower layer. However, the effect of Fe^2+^ on Mn^2+^ removal should be considered. This is mostly due to the dissolution of bioMnO_x_ into Mn^2+^ by Fe^2+^ in the biofilter bed [64], which needs a thicker filter bed or a longer start-up period to remove Mn^2+^. It was shown that the start-up time of a one-stage biofilter required more than 6 months at Fe^2+^ concentrations up to 12 mg/L, compared to 1–3 months at Fe^2+^ values of 7 mg/L. In newly constructed biofilters, mature sand that is coated with bioMnO_x_ or MnOB is usually dispersed as inoculum on the upper filter layer. The redox interaction between high Fe^2+^ and Mn oxides should be carefully monitored.

## 4. The Widespread Application of MnOB-Produced BioMnO_x_

### 4.1. Characterization of BioMnO_x_

BioMnO_x_ is widely used because of its excellent physicochemical properties. Powder X-ray diffraction (XRD) patterns are used to investigate the phase properties. This implies that nearly all bioMnO_x_ has low crystallinity and an amorphous structure, as evidenced by its disordered structure [18,50,65,66,67,68]. When compared to standard JCPDS or PDF cards, these fresh bioMnO_x_ compounds contain one or more characteristic peaks of the model compounds, indicating the precursor of Mn ore minerals [18].

The particle size range of bioMnO_x_ is at the nanoscale [49], but it aggregates to a larger micrometer scale as aging time increases. According to Zhou and Fu [69], the surface area of bioMnO_x_, which ranges from 98 to 224 m^2^/g, is frequently greater than that of chemically synthesized MnO_2_. This is inconsistent with our findings that the bioMnO_x_ produced by a biofilter has a surface area of 39.1 m^2^/g [18], while the as-prepared MnO_2_ shows similar values of 35.41 and 39.29 m^2^/g [70]. The larger the particle size, the smaller the specific surface area. The aggregation of bioMnO_x_ with prolonged aging time is mostly responsible for the reduced surface area in biofilters. Furthermore, crystallinity also increases with aging time, resulting in crystal phase succession [71]. As illustrated in Equations (1) and (2), a larger surface area provides more adsorption sites for pollutants, and a lower crystallinity promotes electron transport in redox processes. That is, using freshly generated bioMnO_x_ for pollution control is preferable.

Elemental valence states can be determined via X-ray photoelectron spectroscopy (XPS). The XPS spectra demonstrate that bioMnO_x_ has multiple valences including Mn^2+^, Mn^3+^, and Mn^4+^. The redox reactions are driven by Mn^4+^ and Mn^3+^, as described in Equations (1) and (2). Their high content suggests that more organic compounds can be attacked, taking more electrons, but the content is affected by MnOB types or cultivation conditions [72]. In any case, the low crystallinity, relatively high surface area, and reactivity of bioMnO_x_ allow it to be employed as an adsorbent, oxidant, and catalyst. It can be concluded that the primary applications of bioMnO_x_ in water environment remediation focus on metal adsorption, the decolorization of organic dyes, and the degradation of emerging pollutants, as summarized below.

### 4.2. Adsorption and Oxidation of Metals

**Figure 4:**
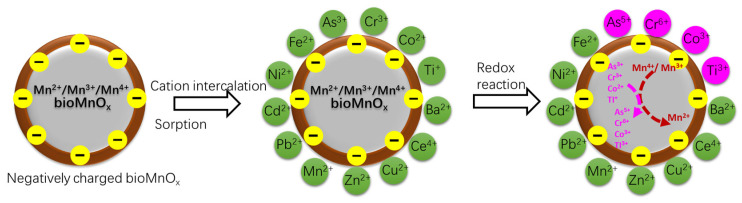
Removal of metals by using bioMnO_x_.

Because of the cation vacancies in the crystal structure, bioMnO_x_ is negatively charged [49]. These negative charges can be compensated for through cation intercalation and sorption, indicating the possibility of heavy metal removal (Figure 4). It is amazing that MnOB can withstand high concentrations of heavy metals. In a culture medium containing 100 mg/L Mn^2+^, Wan et al. [19] evaluated the heavy metal removal capacity of a MnOB consortium. The removal of Mn^2+^ was reduced by 0.4%, 87.5%, 7.7%, and 22.4%, respectively, with the addition of Fe^3+^ of 56 mg/L, Co^2+^ of 56 mg/L, Ni^2+^ of 58.7 mg/L, and Zn^2+^ of 65 mg/L; however, 72.0% of Fe^3+^, 12.6% of Co^2+^, 44.1% of Ni^2+^, and 90.4% of Zn^2+^ could be removed simultaneously. Meanwhile, Cu^2+^ did not inhibit Mn^2+^ removal by MnOB at values ranging from 6.4 mg/L to 96 mg/L until it reached 128 mg/L. Bacterial cell walls, extracellular polymeric sheaths, and bioMnO_x_ can absorb heavy metals, but the capability of the latter is around two orders of magnitude higher than that of the others [73]. To a certain extent, bioMnO_x_, which has a high capacity for adsorbing heavy metals, can protect MnOB from toxicity. Moreover, it indicates that bioMnO_x_ produced by the *Pseudomonas putida* strain MnB1 has a seven to eight times higher adsorption capacity for Pb^2+^, Cd^2+^, and Zn^2+^ than abiotic MnO_x_ (birnessite) [74]. In comparison to abiotic MnO_x_ (todorokite), *Bacillus* sp. WH4-produced bioMnO_x_ has a maximum Cd adsorption capacity that is approximately 2.96 times greater [75]. By modifying the zeolite with bioMnO_x_, the removal of Pb^2+^, Cd^2+^, and Zn^2+^ may also be improved by 36.4–70.5% [76].

Although arsenic (As) is not a heavy metal, it is frequently examined alongside heavy metals. In aqueous environments, As^3+^ and As^5+^ are the two most common forms; however, As^3+^ exhibits greater metal toxicity. In Figure 4, the oxidation of As^3+^ to As^5+^ can reduce the metal toxicity of As. It shows that bioMnO_x_, whose oxidation rate (*k*_1_ = 0.23 min^−1^) is much higher than that of abiotic MnO_x_, can achieve quick As^3+^ oxidation and, subsequently, As^5+^ adsorption [77,78]. Liu et al. [79] indicated that the tolerant concentrations of bioMnO_x_ for heavy metals As^5+^ and Mn^2+^ are 500 mg/L and 120 mg/L, respectively. This explains how biofilters may simultaneously remove Mn and As from groundwater. In a similar way, bioMnO_x_ also transforms the extremely toxic Co^2+^ [80] and TI^+^ [81] into less hazardous metal oxides. On the contrary, the oxidation of Cr^3+^ by bioMnO_x_, which results in the formation of more mobile Cr^6+^, increases the toxicity.

### 4.3. Dye Decolorization

Up to 15% of the dyes used in various industries can be observed in industrial effluents. These dye residues are stable in water and are harmful to living organisms in aqueous environments; thus, they should be removed [82]. Adsorption is believed to be the most tested, environmentally friendly, and effective method [83]. In comparison to chemically produced adsorbents, the synthesis of bioMnO_x_ requires no additional energy input, indicating a low-cost adsorbent. It shows that for each gram of bioMnO_x_ produced from a mixed MnOB consortium, 22 mg of methylene blue and 23.8 mg of crystal violet could be decolorized, respectively [19]. In addition, the bioMnO_x_ produced by *Marinobacter* sp. MnI7-9 has a surface area of 27.68 m^2^/g and a maximum adsorption capacity of 115.61 mg/g for indigo carmine [84].

The combination of adsorption and oxidation promotes decolorization, but the pH conditions must be carefully optimized. This is because the redox processes mediated by bioMnO_x_ are highly dependent on solution pH. Equations (1) and (2) demonstrate that the bioMnO_x_ has a higher oxidation capability at lower pHs. The release of Mn^2+^ from both self-leaching and reduction of the bioMnO_x_ results in secondary contamination and a loss of reactivity under the strongly acidic pH conditions. MnOB can regenerate bioMnO_x_ by the reoxidation of Mn^2+^ [85], but it is ineffective in strongly acidic environments. Due to the resorption of Mn^2+^ by bioMnO_x_, the release of Mn^2+^ can be decreased or absent under weakly acidic pH circumstances. As the pH of the solution increases, the oxidation capacity decreases significantly, and adsorption could become dominant. However, the catalytic reactions mediated by Mn or oxygen vacancies, as described below, may still be involved in the oxidation of dyes.

### 4.4. Control of Organic Contaminants

**Figure 5:**
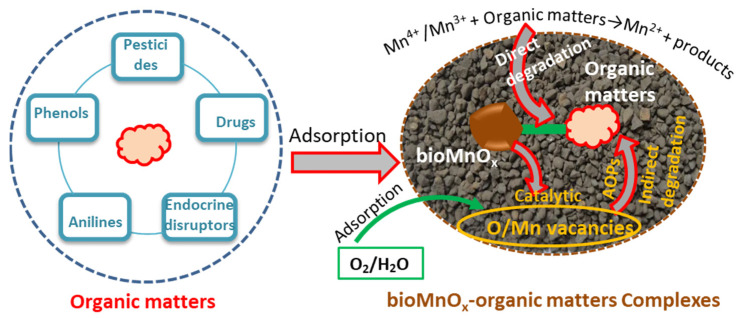
The schematic diagram for the removal of MOCs by bioMnO_x_.

Microorganic contaminants (MOCs) have recently been frequently found in water bodies, indicating possible negative impacts on the ecosystem. These MOCs are stable in water and usually difficult to degrade directly through bioactivity. As a result, physicochemical treatment approaches such as ozonation, advanced oxidation processes (AOPs), and adsorption for degradation or removal have been developed. One of the physicochemical processes is the decomposition of MOCs utilizing bioMnO_x_, which is accomplished indirectly by MnOB. The use of MnOB appears to be an eco-friendly and cheap alternative. To the best of our knowledge, as illustrated in Figure 5, these MOCs that can be degraded include, but are not limited to, antibiotics [68,72], endocrine disruptors [86,87], and pesticides [88]. It implies that the process mediated by MnOB, which has the advantage of combining adsorption and degradation, has a broad spectrum of performance.

Amorphous bioMnO_x_ is more reactive than commercial MnO_2_. As an illustration, the data showed that MnO_2_ barely decomposed carbofuran, whereas MnOB indirectly degraded 90.63% of it under the same conditions [89]. Previous research has shown that the two reactions described by Equations (1) and (2) are commonly acknowledged as MOC degradation mechanisms. In fact, in the liquid phase, the oxygen or Mn vacancies of bioMnO_x_ can catalyze the oxidation of water or oxygen to form reactive oxygen species, functioning similarly to the AOPs (Figure 5). The catalytic capacity increases with worsening crystallinity, which suggests more vacancies. However, the crystallinity of bioMnO_x_ rises as it grows or ages. This could explain why newly formed bioMnO_x_ is more reactive than commercial MnO_2_. The AOP-like degradation process induced by bioMnO_x_ should be studied further, since it has the potential to contribute significantly to degradation. During the degradation of tetracycline hydrochloride by MnO_2_ nanomaterials, Pal et al. [90] observed that the efficiency of tetracycline hydrochloride degradation by MnO_2_ nanoparticles decreased from 66% to 27% and 37% in the presence of the reactive oxygen species scavengers sodium azide and t-butanol, respectively.

## 5. Further Concerns and Challenges

### 5.1. Groundwater Purification

The biofiltration process has been developed and engineered for at least 30 years to remove Mn^2+^ from groundwater. Although Mn^2+^ is found in groundwater alongside Fe^2+^, ammonia, or As, simultaneous removal of these contaminants has been successful. Nitrate concentrations can reach 20 mg/L or greater in some regions, and must be decreased to less than 10 mg/L. According to conventional nitrogen removal theory, carbon sources and anoxic conditions are required in a biofilter for nitrate denitrification. The biofilm can reasonably satisfy the anoxic conditions, since the MnOB has fewer DO needs. However, carbon supplies in groundwater are typically limited; adding carbon sources to groundwater complicates the treatment process and, more importantly, the faster growth of the heterotrophic denitrifying bacteria may take up the required space for MnOB. The simultaneous removal of Mn^2+^ and nitrate, therefore, poses new difficulties for the bioprocess.

Furthermore, the increase of pH of groundwater by over-aeration promotes the chemical oxidation of Mn^2+^. However, it seems that the excessive aeration is unnecessary because it does not substantially accelerate bio-oxidation. Due to the existence of autotrophic nitrogen removal bacteria, even if ammonia increases the DO consumption, the real demand for DO is lower than that of complete nitrification. As a result, determining how to accurately supply DO to save energy is a challenge.

### 5.2. Mn^2+^-Supplied Biofilter Application

A biofilter supplied with Mn^2+^ can be developed for further engineering applications of MnOB. On the one hand, the quality of water bodies, including surface water and groundwater, is complicated by exogenous emerging contaminants. During the biofiltration process, these pollutants, in concentrations ranging from ng/L to μg/L, may be adsorbed or degraded by bioMnO_x_. Importantly, because the amount of bioMnO_x_ determines the efficiency, the fed Mn^2+^ concentrations can be adjusted to ensure it. Regrettably, this has rarely been reported in previous studies, and it should be studied more.

Moreover, bioMnO_x_ is also employed to control high levels of contaminants present in wastewater, such as heavy metals and organic compounds, as illustrated in Figure 4 and Figure 5, but the majority of the relevant study was conducted in flasks. Mn^2+^ is commonly released during the decomposition of large quantities of organic compounds, resulting in the loss of bioMnO_x_ and reactivity. In addition, during the adsorption procedure, the bioMnO_x_ may become saturated. As mentioned, biofiltration is a feasible solution to these issues because it not only re-oxidizes the released Mn^2+^ but also maintains the reactivity of bioMnO_x_ by oxidizing the fed Mn^2+^, allowing for continuous adsorption and degradation in situ, whereas Mn^2+^ must be completely oxidized to avoid secondary pollution.

## 6. Conclusions

Mn^2+^ is commonly found in groundwater at concentrations above the required level for drinking. Hence, it is necessary to remove Mn^2+^ from groundwater. The MnOB-based bioprocess consisting of aeration, biofiltration, and disinfection has been discussed, and the literature survey indicates that Mn^2+^ can be effectively removed without any chemical oxidants. The aeration strength and filter bed should be optimally designed, in accordance with the concentrations of ammonia and Fe, for energy saving and start-up purposes. Further research should focus on the nitrate removal from groundwater by this bioprocess. In addition, a Mn^2+^-supplied biofilter capable of producing bioMnO_x_ can be further developed for water remediation applications such as metal adsorption, dye decolorization, and organic substance degradation.

## Data Availability

Not applicable.
